# Taste and Food Preferences of the Hungarian Roma Population

**DOI:** 10.3389/fpubh.2020.00359

**Published:** 2020-08-04

**Authors:** Judit Diószegi, Péter Pikó, Zsigmond Kósa, János Sándor, Erand Llanaj, Róza Ádány

**Affiliations:** ^1^MTA-DE Public Health Research Group, Public Health Research Institute, University of Debrecen, Debrecen, Hungary; ^2^Department of Methodology for Health Visitors and Public Health, Faculty of Health, University of Debrecen, Nyíregyháza, Hungary; ^3^Department of Public Health and Epidemiology, Faculty of Medicine, University of Debrecen, Debrecen, Hungary; ^4^Doctoral School of Health Sciences, University of Debrecen, Debrecen, Hungary; ^5^Public Health Research Institute, University of Debrecen, Debrecen, Hungary

**Keywords:** Roma, taste preference, food preference, nutrition, diet

## Abstract

**Background:** In Central, Eastern, and Southern Europe, the Roma population is the largest and the most vulnerable and disadvantaged minority. Behind their unfavorable health status, harmful health behaviors, such as unhealthy diet is also supposed to exist.

**Methods:** In the framework of a complex health study, individuals from the Hungarian general (*n* = 410) and Roma populations (*n* = 387) were randomly selected. In the survey portion of the study, sweet, fat, salty, and bitter taste preferences were ascertained by question items measuring taste and food preferences. Preference for sweet vs. salty foods was also analyzed. Questions from the Hungarian version of the European Health Interview Survey were included, to characterize fruit and vegetable consumption and to determine the quantity of sugars added to consumed foods and beverages and the frequency of salting without tasting the food. Data were analyzed using STATA 9.0 statistical software.

**Results:** Roma reported significantly less frequent consumption of fresh fruits (OR = 1.70, 95% CI: 1.22–2.35, *p* = 0.002) and vegetables (OR = 1.74, 95% CI: 1.25–2.41, *p* = 0.001) than the Hungarian subjects. Representatives of the Hungarian Roma population reported adding higher quantities of sugars to consumed foods and beverages (OR = 1.68, 95% CI: 1.10–2.56, *p* = 0.016) and preferred sweet snacks vs. salty ones (OR = 0.53 for salty snacks, 95% CI: 0.37–0.78, *p* = 0.001) and had higher preferences for sweet foods (OR = 1.51, 95% CI: 1.08–2.11, *p* = 0.015). They salted their food without tasting it more often (OR = 2.18, 95% CI: 1.64–2.88, *p* < 0.001). They had lower preferences for bitter tasting raw kohlrabi (OR = 0.56, 95% CI: 0.41–0.80, *p* = 0.001), grapefruit (OR = 0.47, 95% CI: 0.34–0.64, *p* < 0.001), black coffee (OR = 0.50, 95% CI: 0.34–0.73, *p* < 0.001), and dark chocolate (OR = 0.63, 95% CI: 0.46–0.84, *p* = 0.006). No significant results in terms of ethnicity were found on for fatty and salty food preference and other bitter-tasting vegetables.

**Conclusions:** Roma diet may be linked to taste preferences predisposing to unhealthy eating habits. This assumption needs further studies on their dietary behavior. In order to design potentially effective intervention programs targeting Roma populations, it is essential to identify individual, environmental, social, cultural, and behavioral factors and as well as their complex interplay that may affect dietary intake and behaviors.

## Introduction

In the European Union, the Roma population is the largest and most vulnerable ethnic minority population. This minority population faces disadvantages in multiple aspects of life, such as deprived housing conditions, low educational attainment and high unemployment rates throughout Europe (1). All these factors, combined with social exclusion, marginalization, and discrimination [reviewed in (2)], give rise to unequal access to health care services [(1, 3, 4), reviewed in (5)], less favorable self-assessed health status [reviewed in (2)] and presumably a higher risk of early mortality than the majority of populations in Europe [reviewed in (2)]. An estimated 10–12 million Roma people are settled in Europe (6), and Hungary is among the countries (together with Bulgaria, Romania, Slovakia, Czech Republic, and Slovenia) with the highest representation of Roma individuals in the total population. In Hungary, the Roma population represents the largest minority group in the country, with an estimated population size of 876 000 individuals, accounting for 8.9 percent of the total population, and the Roma population is the only ethnic group with a constantly increasing number and ratio in the country's total population (7).

Although many Roma people are partially assimilated into majority societies, most Roma communities have maintained their cultural identity and traditions [reviewed in (2)], which strongly affect their lifestyles and health behaviors (8). Harmful health behaviors, such as unhealthy nutrition, are thought to exist and potentially underlie the unfavorable health status of the Roma population.

Survey results suggest that the Roma diet can be characterized by low and infrequent consumption of fresh fruits and vegetables (3, 9–22) and dairy products (11, 13, 19) and high consumption of fast foods (23, 24), animal fat (16, 18, 25, 26), soft drinks (10, 11, 16, 24, 27), and sweets (16, 27).

By analyzing the lifestyles of 137 young (18–35 years) healthy Slovak subjects of the majority population and 122 young Roma individuals who declared themselves healthy, it was found that Roma individuals consumed fruits less frequently than non-Roma individuals (9). According to face-to-face semi-structured interviews of 302 participants from the Roma population (over 18 years of age) in the South Bohemian Region, participants reported a high frequency of consumption of soft drinks, and a low frequency of consumption of fruits and vegetables (10). A survey conducted in the same region, involving 302 Roma and 298 non-Roma persons, identified low consumption patterns of both fruits and vegetables (17). In the framework of the European project “Health and the Roma Community, Analysis of the Situation in Europe,” promoted by the Fundación Secretariado Gitano, in 2007, 7,604 Roma individuals of all from seven participating European countries (Bulgaria, Czech Republic, Greece, Portugal, Romania, Slovakia, Spain) were interviewed, and it was found that only 28% of Roma individuals consumed fruits and fresh vegetables on a daily basis; in contrast, 36% of them consumed sweets every day (12). Research involving 201 Roma and 201 majority respondents from the county of Rimavská Sobota district of Slovakia found that the Roma diet was characterized by low consumption of dairy products and vegetables (13). Within the framework of a cross-sectional study in 2010, a food frequency questionnaire was administered to a representative sample of 400 subjects aged 15 years from Roma communities in five districts in Albania. The study found infrequent consumption of fruits and milk and moderate consumption of meat and vegetables (14). The estimates of the Household Budget Survey (HBS) of Romania conducted with a sample of ~9,000 dwellings covering the period from 2004 to 2011 suggest that the Roma population has an inferior diet compared to that of non-Roma populations in terms of a lower proportion of dairy product, fruit, and vegetable intake (15). Other Roma studies also suggest high consumption of fatty meats and sweets and low consumption of vegetables and fruits (16). Using the data from the cross-sectional HepaMeta study conducted in 2011 in Slovakia, high consumption of fast foods was identified among the Roma population. The study's sample comprised 452 Roma (mean age = 34.7; 35.2% men) and 403 (mean age = 33.5; 45.9% men) non-Roma respondents (11). Low consumption of vegetables was also reported among 1,200 Hungarian adult Roma respondents (18). A cross-sectional survey in Hungary among 9,040 mothers of neonates was conducted in 2010. The study revealed significantly less frequent consumption of fruits, vegetables, and dairy products among Roma mothers than among non-Roma mothers (19). The proportion of those consuming fresh fruits and vegetables on a daily basis was much higher in the general population than among the Roma population in our previous study (2007), which included data from 969 Roma and 5,072 Hungarian adult respondents (3). In our cross-sectional, questionnaire-based survey conducted in Hungary, which comprised 11-year-old (211 boys and 252 girls) and 13-year-old (205 boys and 247 girls) children living in Roma settlements, the prevalence of consuming sweets and soft drinks at least 5 times per week was 1.5- to 2- times higher among Roma children than among the general Hungarian child population. In contrast to previous findings, this study did not detect any differences in fruit or vegetable consumption between the two samples (27).

The results of two paired health interview surveys that we carried out in the general Hungarian and Roma populations using the same methodology before and after the Decade of Roma Inclusion were compared, and it was clearly shown that the distribution of BMI worsened significantly among younger Roma individuals (in both sexes) between 2004 and 2015, with obesity becoming significantly more frequent (28). These findings support the assumption that unhealthy diet is characteristic of the nutritional profile of the Hungarian Roma population, since obesity is a potential consequence of poor dietary behavior (29).

Eating behavior is a complex trait influenced by both environmental (social, cultural, and community factors) and genetic factors. Individual food preferences are important determinants of food choices and intake (30, 31) and are highly influenced by taste, which is listed among the five main values (taste, health, cost, time/convenience, and social relationships) in the Food Choice Process Model that explains the complex basis of motivations behind dietary practices and food choice decisions (32). Characterizing the taste and food preferences of populations would improve the understanding of food choices and dietary practices.

The aim of the present study was to compare the food choices and taste/food preferences of the Hungarian Roma population to those of the Hungarian general population. The frequent consumption of sweet-tasting soft drinks and fast food with high salt and fat content may be mediated by related taste preferences. To our knowledge, this is the first study designed to characterize food preferences representing certain taste dimensions of the Roma population in comparison with those of the general population. Poor dietary habits and nutrition are important determinants and major modifiable risk factors for non-communicable diseases and may differ in the culturally diverse settings of Roma populations. Exploring individual taste and related food preferences, as well as nutritional attitudes behind dietary choices is essential to aid the development of tailored public health strategies targeting the Roma population.

## Materials and Methods

### Study Design

In the framework of a complex comparative health study, randomly selected individuals from the adult Hungarian general population and Hungarian Roma population living in segregated settlements from Borsod-Abaúj-Zemplén and Szabolcs-Szatmár-Bereg counties were enrolled. The planned sample size was to involve 500 subjects in each study sample. In the survey portion of the study, the questionnaires were administered by nurses in the Hungarian general population and by Roma field workers in the Hungarian Roma population. Data collection was carried out between 17 May and 29 August 2018.

### Samples

#### Randomly Selected Sample of the Hungarian General Population Living in Northeast Hungary

The General Practitioners' Morbidity Sentinel Stations Program (GPMSSP), a population-based registry designed to monitor the prevalence and incidence of chronic non-communicable diseases of great public health importance, has operated in Hungary since 1998 (33). The source population of this registry includes all Hungarian citizens registered by the 59 participating general practitioners. The GPMSSP provided the Hungarian reference sample. The study population involved randomly drawn individuals, who were 20 to 64 years of age, lived in private households and were registered by GPs in Borsod-Abaúj-Zemplén and Szabolcs-Szatmár-Bereg counties (Northeast Hungary). The planned sample size was 25 individuals from 20 randomly selected GP practices in these counties. However, two GPs refused to participate, so the final sample consisted of 450 people from the practices of 18 GPs.

#### Randomly Selected Sample of the Hungarian Roma Population Living in Segregated Colonies in Northeast Hungary

A stratified multistep sampling method was applied to enroll Roma participants from the abovementioned two counties of Northeast Hungary (Hajdú-Bihar and Szabolcs-Szamár-Bereg counties), the region where the majority of Roma colonies are found. Segregated colonies exceeding 100 inhabitants were previously identified by Roma field workers. The ethnicity of the colony population was assessed by self-declaration (34). After verifying the previously created database, 20 colonies were randomly chosen. Subsequently, 25 households were randomly selected from each colony; then, all individuals aged 20 to 64 years in each household were identified, and one person was selected by a random table.

The study was approved by the Ethical Committee of the Hungarian Scientific Council on Health (61327-2017/EKU). Written informed consent was obtained from all participants in each study population in accordance with the Declaration of Helsinki.

### Food and Taste Preference Assessment

In the questionnaire-based portion of the complex health study, questions from the Hungarian version of the European Health Interview Survey (35) were included to characterize fruit and vegetable consumption of the two study groups and to assess dietary preferences by determining the quantity of sugars added to consumed foods and beverages (categories: 0, 1–4, 5–10, >10 teaspoons), as well as the frequency of salting without tasting food (categories: never, sometimes, often, always). Taste preference was ascertained by a food preference questionnaire answered on a five-point Likert scale proposed by Catanzaro et al. (36) (ranging from 1 “dislike extremely”; 2 “dislike moderately”; 3 “indifferent”; 4 “like moderately,” to 5 “like extremely,”; “have not tasted yet” was also given as an option) was administered by asking each individual to rate sweet-, fatty-, salty-, and bitter-tasting food items. Questions were categorized into flavor dimensions and the questionnaire was administered by an interviewer. Sweet, fatty, and salty food preference was investigated by applying single questions with examples of each taste stimulus (sweet: cake, chocolate, ice cream, cookies, wafer bars; fat: bacon, salami, sausage, gravy; salty: salty sticks, chips, crackers), whereas bitter taste was assessed based on the preference of six bitter-tasting food items individually (dark chocolate, black coffee, raw kohlrabi, raw white cabbage, raw cauliflower, and grapefruit). Research suggests that the individual differences in the perception of different bitter tasting food items and beverages may be linked to different genetic polymorphisms, and no such differences were found related to other taste stimuli [reviewed in (37)]. Accordingly, the reason for including individual questions on six bitter-tasting food items was the genetic background of bitter taste perception and preference. Preference for sweet vs. salty foods was analyzed by asking participants to decide between sweet-tasting (cake, cookies, wafer bars, candy) and salty snacks (salty sticks, crackers, pretzels, chips) based on the study of Eriksson et al. on individuals with northern European ancestry (38). This genome-wide association study examined 22 common traits in nearly 10 000 participants and not only identified new genetic associations but also introduced a novel way of collecting data of multiple phenotypes in a large sample of individuals. The associations of sweet taste preference with fruit consumption and of bitter taste preference with vegetable consumption and the relationship between sweet taster status and sweet preference were analyzed as well. Foods to asses taste preferences were chosen according to Hungarian dietary patterns (39) and well-known and available food items in the Roma population (40) (Table 1). Factors influencing taste and food preferences and dietary patterns were registered, such as smoking (41–43) heavy alcohol use (44) and special diet adherence.

**Table 1:** Questionnaire—question items measuring taste and food preferences.

**How often do you consume fruit (fresh/frozen) or fresh-squeezed fruit juices?**						
More than once a day						
Once a day						
4 to 6 times a week						
1 to 3 times a week						
Less than once a week						
Never						
**How often do you consume vegetables (fresh/frozen), salad or fresh-squeezed vegetable juices, excluding potatoes?**						
More than once a day						
Once a day						
4 to 6 times a week						
1 to 3 times a week						
Less than once a week						
Never						
**Which snacks would you prefer, if you had the opportunity to choose?**						
Sweet ones (cake, cookies, wafer bars, candy, etc.)						
Salty ones (salty sticks, crackers, pretzels, chips, etc.)						
**How many teaspoons of sugar do you add to your foods and drinks (tea, coffee, lemonade, cereals, milk, dairy products, pasta, fruits, etc.) on average, per day?**						
None						
1–4 teaspoons						
5–10 teaspoons						
More than 10 teaspoons						
**How often do you salt your food without tasting it?**						
Never						
Sometimes						
Often						
Always						
**Please read the following list of food items and rate how much (on average) you like the specific item**	**Dislike** **extremely**	**Dislike** **moderately**	**Indifferent**	**Like** **moderately**	**Like** **extremely**	**Haven't tasted** **yet**
How much do you like sweet-tasting foods (e.g., cake, chocolate, ice-cream, cookies, wafer bars)?	1	2	3	4	5	9
How much do you like fatty foods (e.g., bacon, salami, sausage, gravy)?	1	2	3	4	5	9
How much do you like salty foods (e.g., salty sticks, chips, crackers)?	1	2	3	4	5	9
How much do you like dark chocolate?	1	2	3	4	5	9
How much do you like black coffee?	1	2	3	4	5	9
How much do you like raw kohlrabi?	1	2	3	4	5	9
How much do you like raw white cabbage?	1	2	3	4	5	9
How much do you like raw cauliflower?	1	2	3	4	5	9
How much do you like grapefruit?	1	2	3	4	5	9

### Statistical Analysis

Data were analyzed using STATA 9.0 statistical software (StataCorp LP, College Station, TX, USA). The Shapiro-Wilk test was applied to test for normality of the age distribution in both study populations. To compare the mean age and sex distribution of the two study groups, the Mann-Whitney *U* and χ^2^ tests were used. Binary outcomes were analyzed with logistic regression, and ordinal outcomes were analyzed with ordered logistic regression. The covariates age, sex, smoking status, and harmful alcohol consumption were controlled in all analyses. The threshold for significance was 0.05. Individuals following special diets were excluded from the analysis.

## Results

The final study sample comprised 410 individuals from the Hungarian general (HG) population and 387 subjects from the Hungarian Roma (HR) population, yielding response rates of 91 and 77.4%, respectively. The mean age did not differ significantly between the two study samples (general Hungarian population: 44.32 ± 12.27, Roma population: 42.78 ± 12.07, *p* = 0.0748). The Mann-Whitney *U*-test was applied to compare the mean age of the two populations since the age distribution did not follow a normal distribution in either study group (Shapiro-Wilk test, *p* < 0.001). The proportion of male individuals in the Roma sample was significantly lower than that in the Hungarian reference sample (0.26 vs. 0.44, *p* < 0.001) according to the χ^2^ test.

### Taste and Food Preferences

In line with previous findings, the Roma participants reported significantly less frequent consumption of fresh fruits (OR = 1.70, 95% CI: 1.22–2.35, *p* = 0.002) and vegetables (OR = 1.74, 95% CI: 1.25–2.41, *p* = 0.001) than Hungarian subjects (for frequency categories see Table 2). The HR population reported adding larger quantities of sugar to consumed foods and beverages (OR = 1.68, 95% CI: 1.10–2.56, *p* = 0.016), preferred sweet snacks rather than salty ones (OR = 0.53 for salty snacks, 95% CI: 0.37–0.78, *p* = 0.001, for frequency categories see Table 3) and had greater preferences for sweet foods (OR = 1.51, 95% CI: 1.08–2.11, *p* = 0.015, for frequency categories see **Table 5**). They salted their food without tasting it more often (OR = 2.18, 95% CI: 1.64–2.88, *p* < 0.001, for frequency categories see Table 4). They had lower preferences for 4 out 6 of the bitter tasting foods, i.e., bitter-tasting raw kohlrabi (OR = 0.56, 95% CI: 0.41–0.80, *p* = 0.001), grapefruit (OR = 0.47, 95% CI: 0.34–0.64, *p* < 0.001), black coffee (OR = 0.50, 95% CI: 0.34–0.73, *p* < 0.001), and dark chocolate (OR = 0.63, 95% CI: 0.46–0.84, *p* = 0.006) (Table 5). No significant results in terms of ethnicity were found for fatty food preference (OR = 1.05, 95% CI: 0.76–1.47, *p* > 0.05), salty food preference (OR = 0.95, 95% CI: 0.68–1.32, *p* > 0.05) or other bitter-tasting vegetables (raw cauliflower OR = 0.90, 95% CI: 0.63–1.29, *p* > 0.05; raw white cabbage OR = 0.83, 95% CI: 0.59–1.17, *p* > 0.05) (Table 5).

**Table 2:** Fruit and vegetable consumption frequencies of the Hungarian general and Roma populations.

**Fruits**	**More than once a day**	**Once a day**	**4 to 6 times a week**	**1 to 3 times a week**	**Less than once a week**	**Never**
HG (%)	20.78	26.27	14.90	28.24	7.84	1.96
HR (%)	15.36	18.81	13.17	29.78	20.69	2.19
**Vegetables**						
HG (%)	9.06	24.80	22.83	31.89	8.66	2.76
HR (%)	12.54	14.11	14.73	32.29	21.32	5.02

HG, Hungarian general population; HR, Hungarian Roma population; OR, odds ratio (covariates: age, sex, smoking status, harmful alcohol consumption).

*The results indicate significantly less frequent consumption of fresh fruits (OR = 1.70, 95% CI: 1.22–2.35, p = 0.002) and vegetables (OR = 1.74, 95% CI: 1.25–2.41, p = 0.001) in the HR than in the HG group*.

**Table 3:** Preference for sweet taste in the Hungarian general and Roma populations.

**Quantity of sugars added to**	**0**	**1–4**	**5–10**	**>10**
**foods and drinks (teaspoons)**				
HG (%)	17.25	69.41	11.37	1.96
HR (%)	5.05	78.86	13.25	2.84
**Sweet vs. salty snacks**	**Salty**		**Sweet**	
HG (%)	51.64		48.36	
HR (%)	33.76		66.24	

HG, Hungarian general population; HR, Hungarian Roma population; vs, versus; OR, odds ratio (covariates: age, sex, smoking status, harmful alcohol consumption).

*Subjects in the HR population reported adding higher quantities of sugars to consumed foods and beverages (OR = 1.68, 95% CI: 1.10–2.56, p = 0.016) and preferred sweet snacks rather than salty ones (OR = 0.53 for salty snacks, 95% CI: 0.37–0.78, p = 0.001)*.

**Table 4:** Frequency of salting food in the Hungarian general and Roma populations.

**Frequency of salting the**	**Never**	**Sometimes**	**Often**	**Always**
**food without tasting it**				
HG (%)	67.84	18.43	5.10	8.63
HR (%)	48.90	22.40	14.20	14.51

HG, Hungarian general population; HR, Hungarian Roma population; OR, odds ratio (covariates: age, sex, smoking status, harmful alcohol consumption).

*HR subjects salted the food without tasting it more often (OR = 2.18, 95% CI: 1.64–2.88, p < 0.001) than the HG individuals*.

**Table 5:** Taste preferences of the Hungarian general and Roma populations measured on a five-point Likert scale.

		**Dislike extremely (1)**	**Dislike moderately (2)**	**Indifferent (3)**	**Like moderately (4)**	**Like extremely (5)**
Sweet foods	HG (%)	3.46	7.69	12.31	47.31	29.23
	HR (%)	3.80	9.81	9.49	28.48	48.42
Fatty foods	HG (%)	3.85	15.00	11.92	50.38	18.85
	HR (%)	6.31	12.93	19.56	34.07	27.13
Salty foods	HG (%)	6.18	12.74	16.99	46.72	17.37
	HR (%)	6.65	12.03	18.99	43.67	18.67
Dark chocolate	HG (%)	28.08	17.31	18.85	21.54	14.23
	HR (%)	35.33	21.45	19.56	17.35	6.31
Black coffee	HG (%)	57.14	19.84	6.75	8.73	7.54
	HR (%)	70.51	19.55	6.09	2.88	0.96
Raw kohlrabi	HG (%)	28.91	7.81	16.80	30.86	15.63
	HR (%)	34.67	14.67	17.67	26.33	6.67
Raw white cabbage	HG (%)	35.90	10.76	17.53	27.89	7.57
	HR (%)	40.00	17.19	16.14	20.35	6.32
Raw cauliflower	HG (%)	48.96	13.69	16.18	17.43	3.73
	HR (%)	50.35	15.97	13.89	13.89	5.90
Grapefruit	HG (%)	28.47	10.77	17.31	31.54	11.92
	HR (%)	49.37	11.71	12.97	19.30	6.33

HG, Hungarian general population; HR, Hungarian Roma population; OR, odds ratio (covariates: age, sex, smoking status, harmful alcohol consumption).

*Roma individuals were characterized by higher preferences for sweet foods (OR = 1.51, 95% CI: 1.08–2.11, p = 0.015), lower preferences for 4 out 6 of the bitter tasting foods, i.e., bitter-tasting raw kohlrabi (OR = 0.56, 95% CI: 0.41–0.80, p = 0.001), grapefruit (OR = 0.47, 95% CI: 0.34–0.64, p < 0.001), black coffee (OR = 0.50, 95% CI: 0.34–0.73, p < 0.001), and dark chocolate (OR = 0.63, 95% CI: 0.46–0.84, p = 0.006). No significant results of ethnicity were found for fatty food preference (OR = 1.05, 95% CI: 0.76–1.47, p > 0.05), salty food preference (OR = 0.95, 95% CI: 0.68–1.32, p > 0.05), and other bitter tasting vegetables (raw cauliflower OR = 0.90, 95% CI: 0.63–1.29, p > 0.05; raw white cabbage OR = 0.83, 95% CI: 0.59–1.17, p > 0.05)*.

### Association of Food Preferences With Vegetable and Fruit Consumption

Higher preference for bitter flavors (i.e., rating raw kohlrabi with high scores on the Likert scale) was associated with higher vegetable consumption in the Hungarian general population sample (OR = 0.79 for lower frequency categories, 95% CI: 0.68–0.92, *p* = 0.003) but not in the Roma population sample (OR = 0.98, 95% CI: 0.84–1.15, *p* > 0.05). Likewise, a higher preference for bitter-tasting raw cauliflower predicted higher vegetable consumption in the HG sample (OR = 0.80 for lower frequency categories, 95% CI: 0.67–0.97, *p* = 0.02), but not in the HR sample (OR = 0.95, 95% CI: 0.81–1.12, *p* > 0.05). Furthermore, similar to the previous results, a higher preference for bitter-tasting raw white cabbage was associated with higher vegetable consumption among Hungarian individuals (OR = 0.75 for lower frequency categories, 95% CI: 0.64–0.90, *p* = 0.001) but not in the Hungarian Roma sample (OR = 0.95, 95% CI: 0.81–1.12, *p* > 0.05). Lower fruit consumption frequency was associated with higher sweet food preference among HG respondents (OR = 1.68 for lower frequency categories, 95% CI: 1.30–2.15, *p* < 0.001) but not among Roma subjects (OR = 0.99, 95% CI: 0.83–1.19, *p* > 0.05).

### Relationship Between Sweet-Liking Status and Sweet Preference

According to a study by Eriksson et al. (38), the question regarding the preference for sweet vs. salty foods identifies people who like sweets. The association of sweet-liking status with sweet preference was analyzed in both study populations. Our results showed that the sweet-liking status was associated with higher sweet food preference in both study populations. The odds ratio (OR) of people who did not like for higher sweet food preference among HG subjects was 0.25 (95% CI: 0.15–0.42, *p* < 0.001), and the association was even more pronounced in the Roma sample (OR = 0.067, 95% CI: 0.04–0.12, *p* < 0.001).

## Discussion

Health disparities by race and ethnicity have become a major focus of public health research, practice, and policy. The seven determinants of health disparities defined by Whitehead (36) comprise freely chosen health-damaging behaviors among others (natural biological variation; transient health advantage of one group due to adopting health-promoting behavior before another group; health-damaging behavior related to severely restricted lifestyle; exposure to unhealthy, stressful living and working conditions; inadequate access to essential healthcare and other basic services; and natural selection or health-related social mobility), which also include the consumption of an unhealthy diet (45). A large number of studies concluded that racial/ethnic and socioeconomic disparities are strongly related to diet quality or diet healthfulness (46–48). Diet-related disparities are defined as “differences in dietary intake, dietary behaviors, and dietary patterns in different segments of the population, resulting in poorer dietary quality and inferior health outcomes for certain groups and an unequal burden in terms of disease incidence, morbidity, mortality, survival, and quality of life.” (49). Since diet is linked to many chronic diseases and conditions, there is growing interest in characterizing the association between taste preference and the prevalence of different risk conditions and major diet-related NCDs, as well as taste and food preferences of specific subgroups of populations.

The European Roma people migrated from India to the Balkans 1000–1100 years ago and currently reside throughout Europe [reviewed in (50)]. In the majority of Central, Eastern, and Southern European countries, this group represents over 5% of the population (51). Their health status is significantly worse than that of the general population, which is strongly related to their unfavorable health behavior characteristics, including eating habits. It seems that Roma people living in Central, Eastern, and Southern European countries experience nutritional problems. They have unhealthy dietary habits, such as irregular and infrequent consumption of fresh fruits and vegetables (3, 9–22) and dairy products (11, 13, 19) compared to consumption patterns in the mainstream populations. Their diet can also be characterized by high consumption of fast foods (23, 24), animal fat (16, 18, 25, 26), soft drinks (10, 11, 16, 24, 27), and sweets (16, 27). It is essential to collect information on the drivers and development of food preferences and specific food choices of the Roma population and to understand the degree to which various factors contribute to health disparities related to dietary patterns to plan and implement culturally appropriate dietary interventions among them.

To our knowledge, this is the first study designed to characterize the taste and food preferences of the Roma population in comparison with those of the general population. In line with previous findings of studies conducted in other European countries, we found that the Roma participants reported significantly less frequent consumption of fresh fruits and vegetables than the Hungarian subjects. In our present study, Roma participants had a higher preference for sweet-tasting foods and reported adding higher quantities of sugars to consumed foods and beverages and salting their food prior to tasting it more often. In addition, they could be characterized by lower preferences for 4 out 6 of the bitter tasting foods, i.e., bitter-tasting raw kohlrabi, grapefruit, black coffee, and dark chocolate and preferred sweet snacks rather than salty ones.

Low consumption of fruits and vegetables can be linked to the low socioeconomic status of Roma people, but food preferences representing certain taste modalities may also be influenced by other (e.g., cultural, genetic) factors. The genetic background of bitter taste preference is well-established, and research suggests the effects of genetic variations on other taste modalities as well [reviewed in (37)]. It is reasonable to suppose that genetic diversity underlies the differences in the taste-related food preferences of Roma people, since interethnic differences were found in certain genetic polymorphisms between the Roma and majority populations in Europe [reviewed in (52, 53) and discussed in depths in (54, 55)].

However, the effects of environmental factors on food preferences among the Roma population are certainly not negligible and include *in utero* exposure, early postnatal experiences, parental feeding practices, family factors (social, economic factors) and the wider contexts of the environment. Research shows that prenatal experiences with food flavors during pregnancy and/or lactation through the mother's diet partly define the preference of the offspring (56, 57). Early childhood is a critical period to establish food preferences and influence diet quality throughout life [reviewed in (58)]. According to semi-structured interviews among Slovak Roma living in Tinsley, Sheffield, bottle feeding Roma babies are often fed with sugary tea (59). Continuous exposure to foods or unfamiliarity with certain flavors and taste factors can differ greatly between families, cultures and ethnic groups. It is suggested that individuals with prior experience and higher consumption frequency with a food item generally express a greater liking compared to those with no prior exposure (60–62). The timing and repeated intake of bitter-tasting fruits and vegetables should be implemented during childhood at home and in childcare settings [reviewed in (58, 63, 64)]. The lack of regular consumption of these vegetables among Roma individuals; parental practices of feeding unhealthy food items, such as soft drinks and sweet foods and inadequate dietary diversity may also result in lifelong unhealthy dietary preferences. These preferences may lead to excess sugar and salt consumption and low intake of certain fruits and vegetables because when consumers focus on taste rather than health implications of their diet in general, they make unhealthier food choices (65).

Higher preference for bitter-tasting raw kohlrabi, raw cauliflower and raw white cabbage was associated with higher vegetable consumption in the HG population sample but not in the HR sample. Lower fruit consumption was associated with higher sweet food preference among HG respondents but not among Roma subjects. In our current research, we did not obtain information on the type or taste of the vegetables (bitter/sweet) (66) and fruits (sweet/sour) consumed (67) therefore, the interpretation of the association between preference and vegetable/fruit consumption is very limited. A possible explanation may be the diet diversity of the Roma population, which is inferior to that of the mainstream populations of Europe (15). Bitter-tasting vegetables are probably less frequently consumed by the Roma population than by the majority populations of Europe. In this study we did not investigate availability and accessibility of fruits and vegetables, but it is clearly shown by previous studies that due to material deprivation, Roma may even undergo difficulties accessing healthy foods (26, 59, 68).

Our research suggests that the unhealthy diet of the Roma population may be linked to their taste preferences. Excessive sugar intake is responsible for the development of certain metabolic diseases, such as diabetes and cardiovascular diseases [reviewed in (69)]. According to the meta-analysis of Wang et al. (70) higher consumption of fruits and vegetables is associated with a lower risk of all-cause mortality, particularly cardiovascular mortality. Adiposity has been shown to be a critical risk factor in the course of the development of insulin resistance, type 2 diabetes [reviewed in (71)] and atherogenic dyslipidemia, which occurs with low-fat, high-carbohydrate diets and increases the risk of coronary heart disease (72). The correlation of salt (sodium) intake with blood pressure has been confirmed [reviewed in (73)]. Therefore, taste preference clearly plays an important role in determining health status. The environmental factors influencing food choices (including possible and continuous exposure to certain tastes and availability of different food items) [reviewed in (74)], should be addressed when considering the dietary habits of the Roma people.

The obvious limitation of the current study is that the Roma study population was not representative of the overall Roma population living in Hungary. The sample was randomly selected from Hungarian Roma individuals living in segregated colonies in Northeast Hungary. Roma individuals who were, to various degrees, assimilated with the general Hungarian population were not included in the analysis due to the nature of the study design. Many people are reluctant to self-define their ethnicity as Roma, and the reference sample of the Hungarian general population included some people who are Roma. It is also important to note that the age range of subjects in both populations was limited to 20–64 years. Another possible limitation is the different response rates of the two study samples. The response rate of the Hungarian sample was 91%, while it was less favorable (77.4%) among the Roma population. Survey response rates of Roma surveys differ to a great extent (28–96.9%) (3, 27, 75–78), and can be lower among Roma populations than among the majority populations included in research (27, 75, 78). This phenomenon can be due to the lack of financial incentives for participation and motivation of Roma to be involved in free medical examinations and to the existing mistrust of segregated Roma toward interviewers. To address the latter issue, Roma field workers were involved in our study; however, higher response rates were not achieved. Although the response rate of Roma individuals in our study was lower than that of the reference sample, it is quite high if we compare it with the majority of Roma studies (75–77). Furthermore, sweet, fat, and salt preference was investigated by applying only single questions, whereas bitter preference was assessed with questions for six bitter-tasting food items individually. Considering that food preferences may be quite specific, using single questions to identify preferences for sweet salty and fatty food items is a limitation of our study. Nevertheless, we have adapted these questions from a two-center, cross-sectional Italian study, which tested some preferences with single questions, so it makes our results comparable with findings obtained in an international study (79). However, it was important to include several questions for bitter preference since there are several receptors for bitter taste (not the case for sweet taste) [reviewed in (37)]. The “sweet-liking status” identified by the question of Eriksson et al. (38) was associated with sweet food preference in our samples. Therefore, the inclusion of this simple question may be suggested in surveys in which only a limited number of questions can be used. We also consider it necessary to mention that we do not know, whether any medication affecting perception was used by the study participants.

To design potentially effective nutrition intervention programs, it is important to identify the individual, environmental, social, cultural, and behavioral factors and their complex interplay that may have an effect on dietary intake and dietary behaviors. All the barriers need to be addressed, such as social and cultural symbolism of foods, poor taste and cost of healthy foods and lack of information (80). Strategies to reduce diet-related disparities include individual-level approaches (targeted nutrition education, dietary counseling, and intervention programs; a focus on cultural and environmental attributes; the use of innovative approaches and novel channels for delivering nutrition education and interventions; the identification of strategies for improving recruitment/retention in intervention) and societal-level approaches (increasing healthy food options in low-income neighborhoods; increasing healthy options in schools; creating environments to encourage physical activity; addressing other barriers to behavioral change) (49). Roma individuals need information on basic nutrition topics and should be encouraged to build a healthy diet and programs targeting this population must be culturally relevant and sensitive to their lifestyle (80). Addressing the problem of food accessibility among Roma people is essential.

Knowledge of the effect and extent to which genetic factors influence the development of individual taste preferences and eating patterns is also important for public policy actions addressing dietary behaviors. The role of genetic polymorphisms in taste perception and taste preferences among the Roma population should be elucidated by future studies since such genetic association studies have not yet been conducted. Regardless, it is important to emphasize that further research is needed on the dietary habits of Hungarian Roma people.

## Data Availability Statement

The datasets generated for this study are available on request to the corresponding author.

## Ethics Statement

The study was approved by the Ethical Committee of the Hungarian Scientific Council on Health (61327-2017/EKU). The patients/participants provided their written informed consent to participate in this study.

## Author Contributions

JD was involved in the development of the questionnaire regarding taste and food preferences, analyzed data, and wrote the manuscript. PP took part in the creation of the database and in the coding and sorting process of data. ZK and JS were involved in the design of the complex comparative health survey and data collection. EL participated in the manuscript preparation. RÁ took part in all steps of the development of the complex comparative health survey, guided the writing of the manuscript, and was involved in finalizing it. All authors contributed to the article and approved the submitted version.

## Conflict of Interest

The authors declare that the research was conducted in the absence of any commercial or financial relationships that could be construed as a potential conflict of interest.
